# Analysis of dynamic contact network of patients with COVID-19 in Shaanxi Province of China

**DOI:** 10.1038/s41598-021-84428-x

**Published:** 2021-03-01

**Authors:** Zhangbo Yang

**Affiliations:** 1 School of Humanities and Social Science, Xi’an Jiaotong University, Xianning West Road, Mailbox 200, Xi’an, 710049 China; 2 Institute for Empirical Social Science Research, Xi’an Jiaotong University, Xi’an, China

**Keywords:** Health policy, Viral infection, Scientific data

## Abstract

The spread of COVID-19 is closely related to the structure of human social networks. Based on 237 cases, by using epidemiological retrospective statistics, data visualization, and social network analysis methods, this paper summarized characteristics of patients with COVID-19 in Shaanxi, China, and analyzed these patients’ dynamic contact network structure. The study found that there are many clustered infections through strong ties, about one-third of cases are caused by relatives' infection. In early stages of the epidemic, imported cases were the most, and in the later stages, local infection cases were the most. The infected people were mostly middle-aged men. Symptoms of imported cases occurred on average of 3 days after they arrived, and medical measures were taken 5 days later on average. All cases showed symptoms in less than 2 days on average and were then taken to medical treatment. The contact network can be divided into multiple disconnected components. The largest component has 12 patients. The average degree centrality in the network is 0.987, average betweenness degree is 0, average closeness degree is 0.452, and average PageRank index is 0.0042. The number of contacts of patients is unevenly distributed in the network.

## Introduction

Coronavirus Disease 2019 (COVID-19) is the third wave of coronavirus outbreaks in the twentieth century^1^. The outbreak of the epidemic in Wuhan, China in early December 2019 caused concern among people all over the world. The World Health Organization has defined the epidemic as a public health emergency of international concern.

Since the outbreak, analyses of COVID-19 are mostly based on epidemiology, virology, and medicine, involving case analysis of patients, construction of infection models, gene sequencing, clinical diagnosis, etc.^2–6^. Epidemiological research is mainly based on patient cases to predict trends of the epidemic in order to better control the spread of the virus^7^. Virology analyzes the biological structure of coronavirus in preparation for vaccine development^3^. Medicine research focuses on the diagnosis and treatment of disease symptoms^8^. However, very few studies analyze the whole network structure and characteristics of virus transmission from the perspective of social networks. Moreover, existing research is also insufficient in mining information behind the public case reports. Integrating multiple case reports can outline the movement trajectory and contact network of specific patients, which may help us better understand the dynamic transmission network of the epidemic.

In human society, the spread of disease is affected by three key factors: the biological structure of the virus, the social structure of the society, and individuals’ cognition regarding the virus. Individual cognition of the virus, which determines the effectiveness of precautionary measures^9^, is of great importance. For example, unrealistic optimism would hinder people’s risk awareness during pandemic^9^. The virus’s transmission route reflects the movement of people and the composition of their social networks. Virus carriers' contact and interaction with each other form a network that is called a contact network. Nodes in the network represent people and edges represent various types of contact relationships, including both strong ties in families and between strangers^10, 11^. Through these contacts, the virus can spread in human society. Analysis of the virus contact network is the key to understand the spread of disease, helping to clarify the transmission route of the virus, and is also an important basis for precautionary measures. Studies show that virus immune strategies based on contact networks are superior to random immune strategies^12^. We also need to note that the susceptibility and infectiousness of the population are the most fundamental elements affecting the spread of the virus. Only in combination with the biological aspects of infection can the network better and more comprehensively capture the dynamic spread of the virus. Most of the current studies are based on epidemiological retrospective studies of patients with COVID-19, and lack of analysis on dynamic contact networks of patients.

Many studies have analyzed the spread of various types of viruses, such as Sexually Transmitted Disease (STD)^10^, Acquired Immune Deficiency Syndrome (AIDS)^13^, and the Plague^11^, from the perspective of social network. Different virus has different transmission ways, making their contact network significantly different. For example, Human Immunodeficiency Virus (HIV) is transmitted through sexual contact, blood contact, and mother-to-child contact, the network structure is relatively simple and sparse. As a comparison, for viruses like the COVID-19, the contact network structure is very complex and dense.

The contact network of many diseases is a small-world network^14^. This type of network has two characteristics. One is that many local connections will form dense local clusters, the other is that there are occasional long-distance connections that can span regions and groups and connect different local clusters^15^. In such a network structure, even if each person has a limited number of contacts, the virus can still quickly spread to the entire network, causing an outbreak of disease^10^. This feature leads to the virus to spread beyond the original infection place, and cause synchrotron oscillation of disease outbreaks in different places^14^. Unlike contact network, the spatial network of viruses has distinct scale-free network features^16^. Studies of contact networks for Severe Acute Respiratory Syndrome (SARS) have shown that only differences in network structure can significantly change the curve of the outbreak^17^. For viruses with a basic regeneration number less than 2, changes in the network structure also have a significant impact on the spread of the disease^18^.

In epidemiological studies of COVID-19, the Lancet first published 41 cases, with a brief description of demographic characteristics of the patients^19^. The median age of those patients was 49, and 66% of them had seafood market contact history in Wuhan, China. There was a case of family cluster infection in their samples. Another analysis of 99 patients showed that the average age of patients was 55 years old and the standard deviation was 13 years old, including 67 males and 32 females, 49 of whom had a history of South China seafood market exposure^20^. Further analysis of 835 cases in Hubei Province of China showed that the average age of the patients was 49 years old, the male to female ratio was 2.7 to 1, and the fatality ratio was 2.9%^7^. Relevant modeling shows that the basic regeneration number of COVID-19 is between 2–3, which is smaller than that of SARS^2, 4^. Summarizing the existing research, the early outbreak of the virus was based on the clustered infection in the South China Seafood Market in Wuhan. The group with the highest virus infection rate was men who were older than 55 years. The virus is mainly transmitted through droplets and contact. Family-based cluster transmission is more common^2, 5^. These studies on the biological properties of COVID-19 are also the basis for our network research and analysis.

This study collected all confirmed cases published in Shaanxi, China from January 23, 2020, to February 16, 2020, a total of 237 cases. First, this paper analyzes the epidemiological characteristics of patients with COVID-19 in Shaanxi Province and then studies the transmission route and contact network.

## Methods

### Methods

I mainly adopt two research methods. One is the descriptive epidemiological research method, to digitally portrait the COVID-19 infected people in Shaanxi Province and analyze their epidemiological characteristics. The other is the social network analysis method to build and visualize patient's contact network and calculate relevant network indicators, including degree centrality, closeness centrality, betweenness centrality, and PageRank index. I construct a dynamic network for corresponding analysis.

### Data

All the samples of this study are officially announced cases of the Health Committee of Shaanxi Province, China, during period from January 23 to February 16, 2020. Cases, later on, are not included because its growth slowed down significantly after February 16th. The paper analyzes and encodes the text of each report.

All methods were carried out by relevant guidelines and regulations. The study received approval from the Ethics Committee of Xi’an Jiaotong University Health Science Center (No. 2020-1217). I collected only the anonymous data. The Ethics Committee of Xi’an Jiaotong University Health Science Center waived the need for informed consent as part of the study approval since this was a retrospective data analysis.

### Measures

First, I coded the demographic characteristics and case characteristics of each case by its published information, including gender, age, household registration, place of infection, time to arrive in Shaanxi, time when symptoms occur, time to visit a hospital or be in quarantine.

Second, I coded the routes of infection no matter it is a stranger tie, a strong tie, or a weak tie. Social networks of human beings consist of these three types of ties^21^. Strong ties refer to close friends, acquaintances, and family members who have frequent daily contact, deeper affection intensity, and high-level trust. Weak ties are those connections of lower contact frequency, lower affection intensity, and lower-level trust than strong contacts. Data from the Shaanxi Health and Medical Commission did not fully specify people infected by which kind of a tie, so I coded data according to the following rules: If the case shows the infection was from their family members or close contacts, then speculates that there is a strong tie. If the case is not clearly stated, I coded it based on the household registration, work conditions, and travel conditions stated in the report. Therefore, the three routes of infection are not mutually exclusive in the data presented. If it is possible to be infected through one route, the code is 1, otherwise 0. For example, if the patient has lived and worked in Hubei Province for a long time, and also got infected in Hubei, I would speculate that she/he might be infected through three ways: strangers, weak ties, and strong ties. But if the patient only stopped by Wuhan when the train returned to Shaanxi, I would speculate that she/he was only likely to be infected by a stranger. Also, I counted whether patients had a relative infection.

Third, the Shaanxi Health Commission's data lists the patient's contacts with each other. Based on the case data, I construct a patient contact matrix in chronological order, visualize the daily dynamic network, and calculate the corresponding network indicators.

## Results

### Basic characteristics of patients with COVID-19 in Shaanxi Province

Table 1 summarizes the frequency and percentage of related variables, which can outline the basic situation of the cases. Specifically, there are slightly more male cases and slightly more cases got infected inside Shaanxi Province. About 59% cases may be infected by strangers, and about 60% may be infected through weak ties such as general colleagues and friends. About 74% cases may be infected through strong ties such as close friends and relatives. 37% cases’ relatives were also infected, which indicates that there are more clustered infections in the region.

**Table 1:** Descriptive statistics of cases with COVID-19 in Shaanxi Province, China.

Gender	Frequency	Percentage (%)	Infected place	Frequency	Percentage (%)
Female	108	45.57	Inside Shaanxi Province	124	52.32
Male	129	54.43	Outside Shaanxi Province	113	47.68
Total	237	100	Total	237	100

Is there a possibility of being infected by a stranger?	Frequency	Percentage (%)	Is there a possibility of being infected through weak ties?	Frequency	Percentage (%)
Yes	140	59.07	Yes	143	60.34
No	97	40.93	No	94	39.66
Total	237	100	Total	237	100

Is there a possibility of being infected through strong ties?	Frequency	Percentage (%)	Whether any relatives are infected?	Frequency	Percentage (%)
Yes	176	74.26	Yes	87	36.71
No	61	25.74	No	150	63.29
Total	237	100	Total	237	100

**Figure 1:**
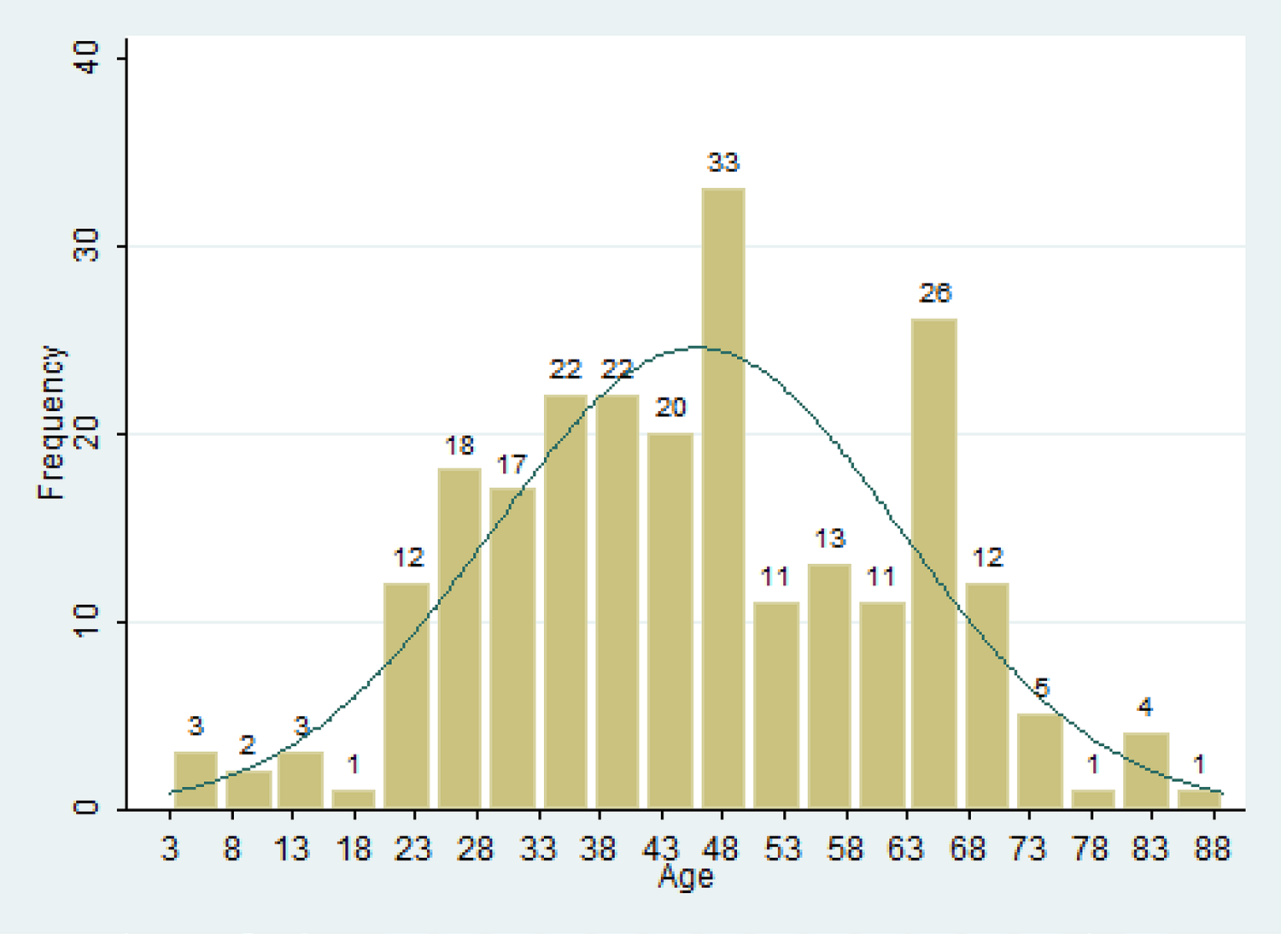
Age distribution of COVID-19 Cases in Shaanxi Province, China.

The frequency distribution of age conforms to normal distribution (Fig. 1). Among them, 48-year-olds have the most infections, and young people age 16 to 20 have the least infections. In general, the number of infections rises sharply above 22.

**Figure 2:**
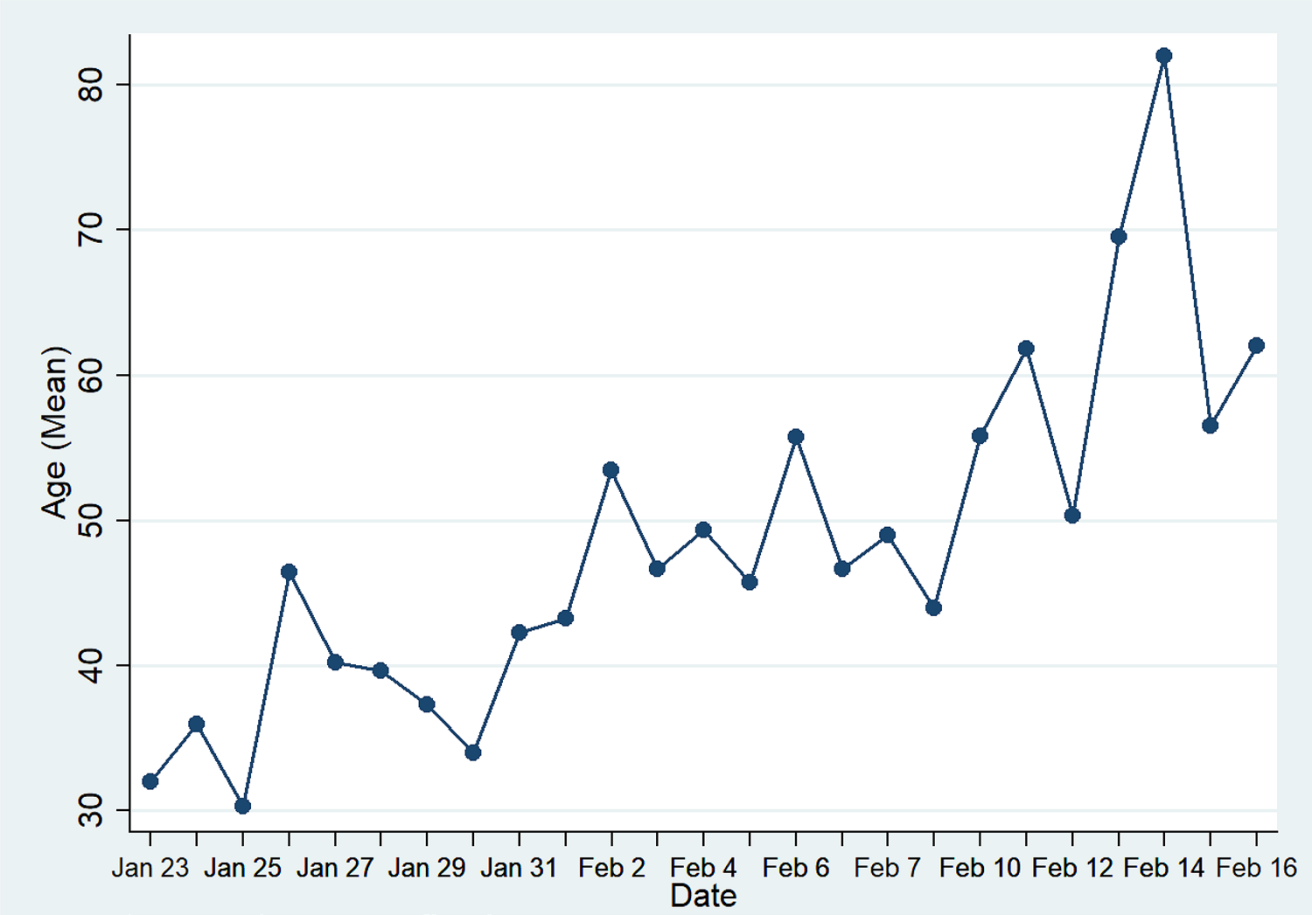
Mean Age of COVID-19 Cases in Shaanxi Province, China.

Figure 2 shows the average age of infected persons increased significantly as time goes. By February 14, the cases’ average age becomes more than 80 years old. The infected people in later stages are older and weaker people who have weak transmission ability, and infection of those young and middle-aged with strong transmission ability was controlled.

**Figure 3:**
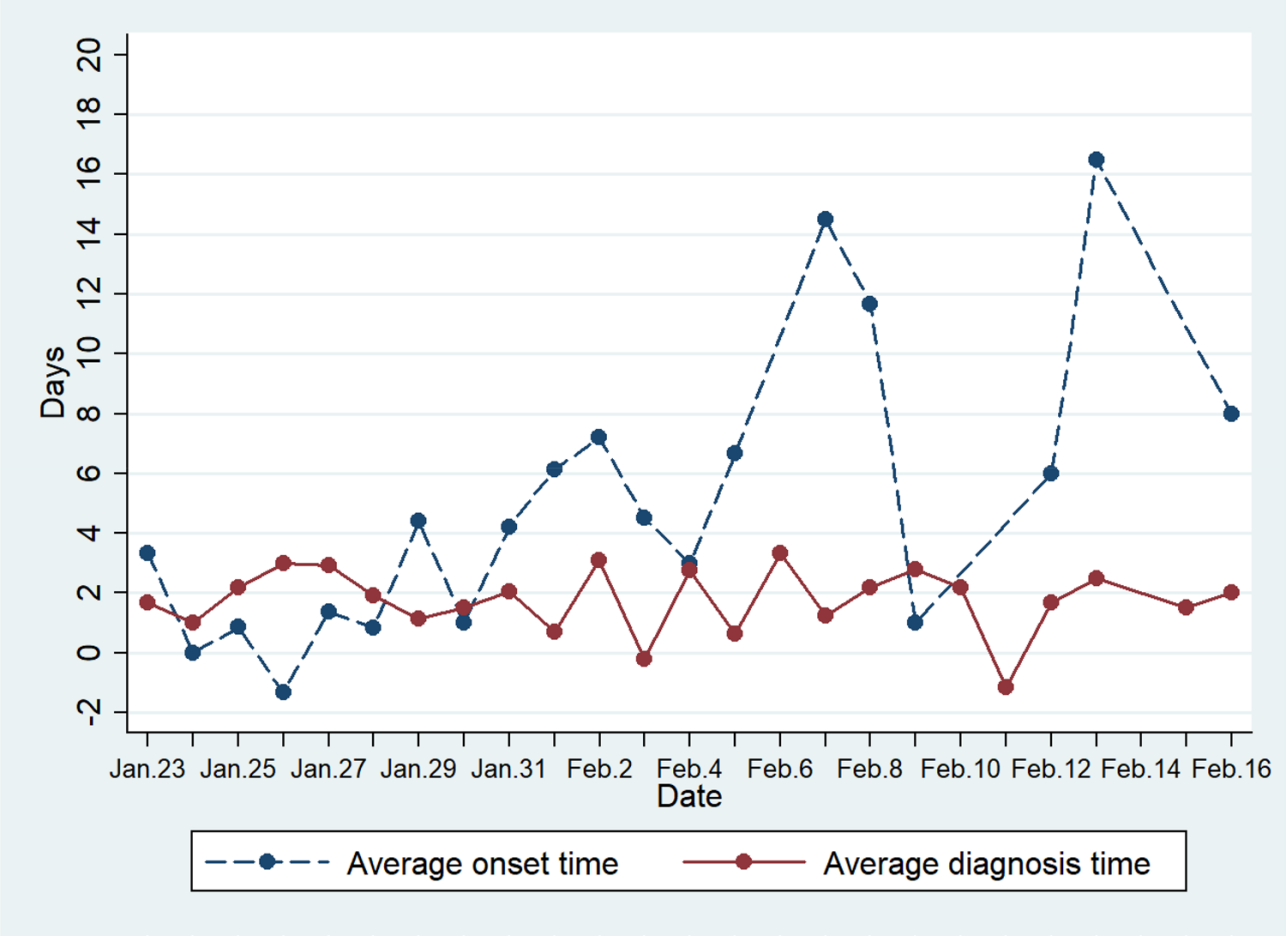
Changes in the average onset and diagnosis time of COVID-19 cases in Shaanxi Province. Average onset time refers to the time when symptoms occur in imported cases after they reach Shaanxi. Average diagnosis time refers to the time taken for all cases to take medical measures after symptoms appear. Both calculations are based on daily published cases.

Table 2 and Fig. 3 show the average onset time of imported cases after arriving in Shaanxi, and the average interval of taking relevant medical measures after symptoms (such as cough, fever, etc.). According to Table 2, the average age of all cases was 46 years old, with the youngest 3 years old, and the oldest 89 years old (this case died in March; the only died case in Shaanxi). For imported cases from other regions in China, they developed symptoms on average of 3 days after arriving in Shaanxi. But the onset date between cases ranges a lot. One case showed symptoms as early as 5 days before arriving in Shaanxi, and another one did not show any symptom until 19 days after arrived. After imported cases arrived in Shaanxi, they went to the clinic or were quarantined after an average of 5.4 days. Case with the shortest interval had a history of visiting doctors one day before arrival, case with the longest interval, however, did not go to the hospital or be quarantined until 17 days after his arrival. After the onset of symptoms, the average time to take relevant treatment was 1.6 days. Case with the shortest time was quarantined 8 days before the onset of symptoms, and case with the longest time did not go to a hospital until 14 days after symptoms appear. No doubt that the latter case has a higher risk of virus transmission.

**Table 2:** Statistics related to the onset time of COVID-19 cases in Shaanxi Province, China.

Variables	Case number	Mean	S.E	Minimum value	Maximum value
Age	237	45.90	16.58	3	89
Symptom onset date minus arriving in Shaanxi date (days)	94	3.489	4.560	− 5	19
Diagnosis/quarantine date minus arriving in Shaanxi date (days)	86	5.488	3.846	− 1	17
Diagnosis/quarantined date minus Symptom onset date (days)	178	1.607	2.973	− 8	14

As time goes, the average onset time of symptoms has a tendency to increase (Fig. 3), which means that the later imported cases mostly have a longer incubation period. Therefore, they were not detected in the early stage. At the beginning of the epidemic, Shaanxi Province adopted measures such as quarantine for cases with short incubation periods. It can be seen that with the change of time, the average diagnosis time has a decreasing trend, which means that the later prevention and control measures are taken on time. Quarantine reduces the risk of virus spread caused by the incubation period.

**Figure 4:**
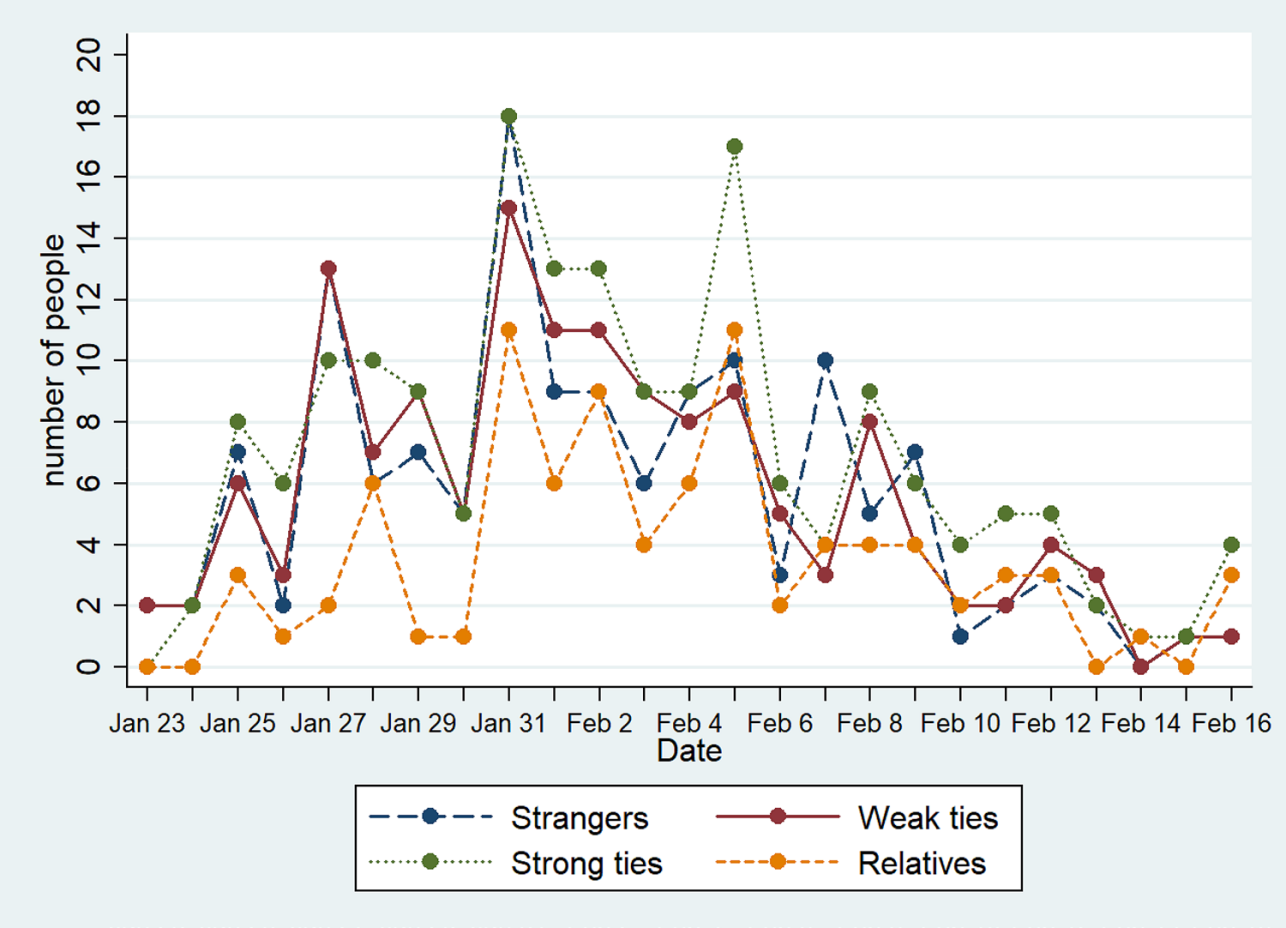
Social network infection route of patients with COVID-19 in Shaanxi Province. Strangers refer to how many of the cases announced that day may have been infected by strangers. Weak ties indicate how many people may be infected by weak tie contacts. Strong ties refer to how many people may be infected by strong tie contacts. Relatives refer to whether any of the patient's relatives are infected.

The transmission route of COVID-19 is mostly through respiratory droplets and individual contact. From the perspective of social network, infection occurs in three kinds of connection: strangers, weak ties, strong ties. Different types of ties can largely affect the number of infections (Fig. 4). My main concern is the proportion of each infection route. Strong ties infection route has higher infection possibility than other routes, showing in Shaanxi is that the cases are mostly cluster infections. There was also an outbreak of stranger infections on February 7, in Xi'an Duocai Shopping Center, where customers and businesses were infected, and most of them were strangers or weak ties. Correspondingly, it also shows that the clustered strong ties infection is the way that needs to be controlled in the epidemic prevention and control, which is consistent with the conclusions of various previous studies.

### Dynamic contact network of COVID-19 cases

**Figure 5:**
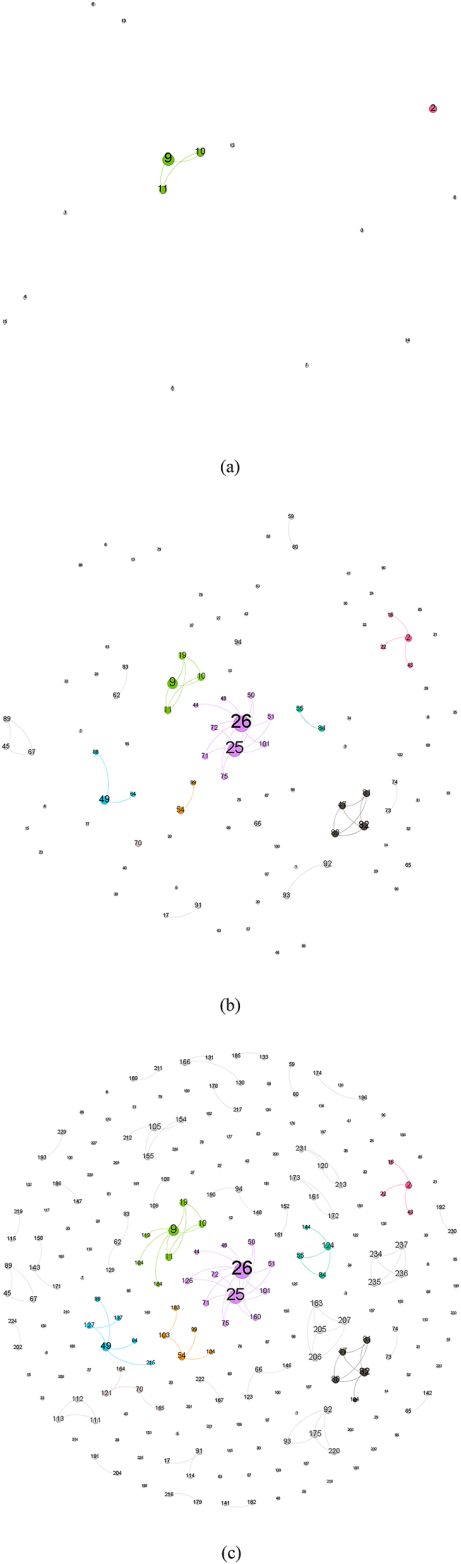
Dynamic contact network of cases with COVID-19 in Shaanxi Province. In the network, nodes represent cases, edges represent transmission routes between cases. The larger the node size, the greater the number of the case connected with. The number on each node is the case number. The larger the number, the later the case is diagnosed. (**a**) Contact Network on January 25, 2020. (**b**) Contact Network on February 1, 2020. (**c**) Contact Network on February 16, 2020.

Figure 5 shows the dynamic contact network of patients with COVID-19 in Shaanxi Province. I intercepted three-time points to present the network structure: early stage network (January 25), intermediate stage network (February 1), and late stage network (February 16).

The early contact network was relatively sparse, most cases could not be identified as infection source. At this time, the largest cluster (component) was composed of three cases, number 9, 10, and 11, and their infection places were all in Wuhan. In the middle period, cluster-shaped infections have appeared, several major clusters form. Case 26′s cluster expanded into the largest one in the late stage. The later network was divided into multiple clusters, the largest one was caused by number 25 and 26 illustrated in the middle of the picture. They were a couple, natives of Shaanxi, who had symptoms after return from Wuhan by driving. They went to local hospitals 5 days after they had symptoms. Case 160 at the late stage could also be traced to this cluster. However, there were fewer new clusters in the later period, which indicates that the control measure of virus transmission is better. In the late period, only cases 234–237 formed a fully-connected component. They belong to one family. There are still many unconnected cases in the network, most of which are imported cases. In addition, when considering the age of the patients, it can be observed that there are some clusters where the chain of infection reflects a gradual increase in the age of the infected individuals. For example, in cluster 9–10–11 of Fig. 5c, the initial infected individuals were a family returning from Hubei, with a couple aged 45 and 46 years old, respectively. The two later infected case 104 who was an elderly member of the family.

**Figure 6:**
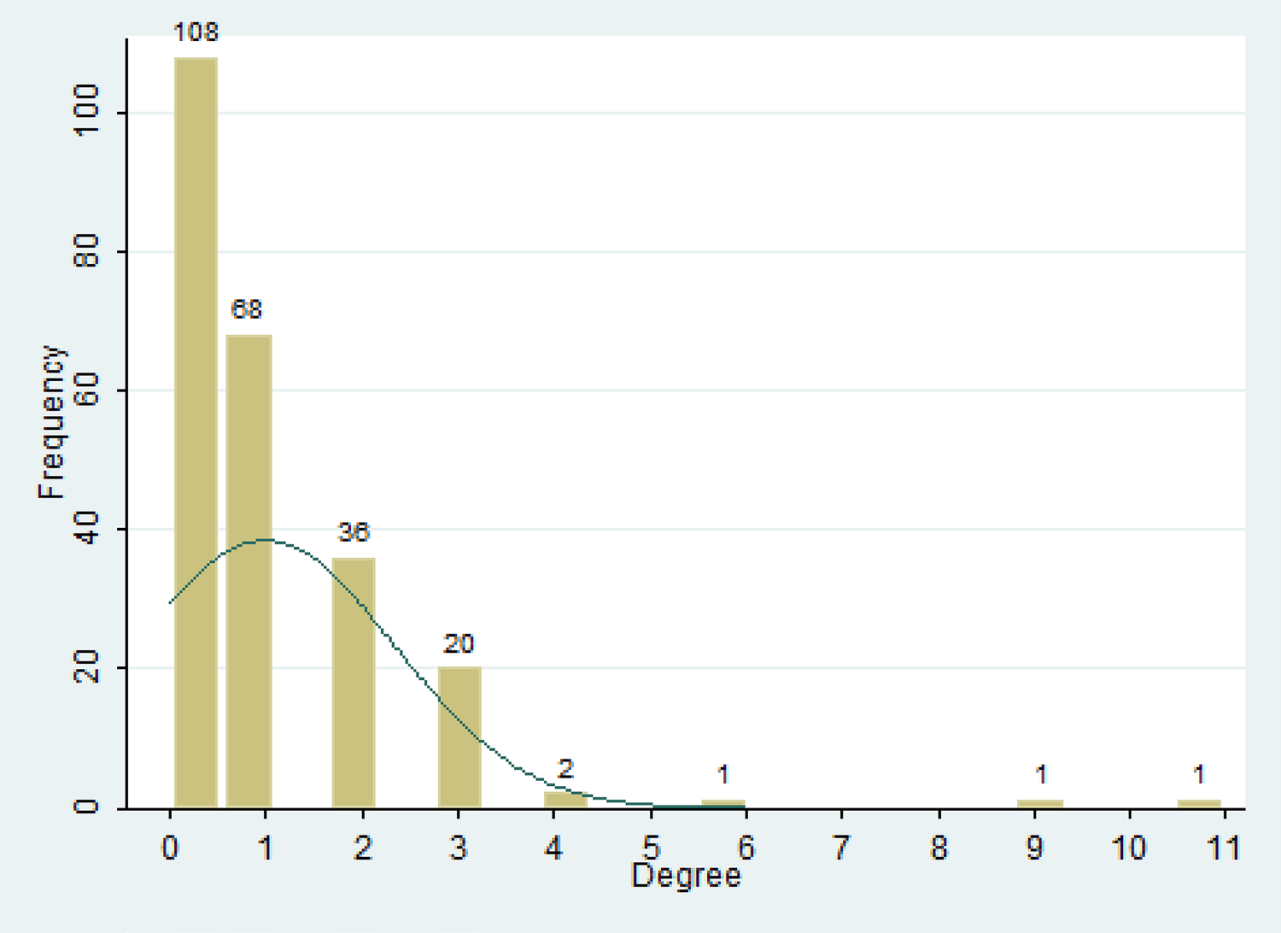
Distribution of degree centrality of contact network of COVID-19 cases in Shaanxi Province.

Table 3 reports four centrality measurements of the contact network. Degree centrality expresses that, on average, how many other cases the focal case has contact with, which is slightly larger than the basic regeneration number. The average centrality degree is less than 1. The smallest degree is 0, the largest is 11, indicating that the patient (case 26 in Fig. 5) was contacted with 11 other patients. Closeness centrality indicates the closeness of the case with other cases. A higher value indicates faster transmission between cases and fewer intermediate cases. The average value is 0.452, which is slightly higher closeness centrality, indicating that the infection is mostly cluster infection. Over time, many aggregated sub-networks are formed, but the network is not fully connected under the action of prevention and control measures (Fig. 5). Betweenness centrality indicates the level of the case as an intermediary in the spread of the virus. The average value is 0, which is very low, indicating that there are few chain transmissions. According to Fig. 5, we can see the transmission mode is mostly one-to-many, that is, A-B, A-C transmission. The PageRank index measures the centrality of the case’s position in the whole contact network. The average value is 0.0042, which is very low. But the maximum value is 0.0228, which indicates that the degree of connection is unevenly distributed among patients. Highly infectious persons can cause a major outbreak. The spread of the Ebola virus is being associated with these super disseminators^22^. In Fig. 6, a small number of people have a higher degree of centrality. But only three cases have a centrality degree greater than 5. Most cases’ degree centrality is zero. This means that although the degree distribution of cases in Shaanxi Province is uneven, the highest number of contacts is low, and there is no super disseminator.

**Table 3:** Statistics of the contact network of COVID-19 cases in Shaanxi Province.

Variables	Case no.	Mean	S.D	Minimum value	Maximus value
Degree Centrality	237	0.987	1.351	0	11
Closeness Centrality	237	0.452	0.440	0	1
Betweenness Centrality	237	0	0.0001	0	0.0012
PageRank	237	0.0042	0.0035	0.001	0.023

The value of closeness centrality and betweenness centrality is in normalized form.

## Discussion

From a network perspective, the quarantine measure can disconnect the virus contact network, making the whole network structurally fragile. Therefore, finding the source of infection and adopting quarantine can quickly control the virus spread. Based on the results, the epidemic prevention policy should pay special attention to cluster infections, prevent cases from forming a giant component in the contact network, cut off the transmission. Of course, the quarantine policy based not only on contact network alone, but also on detection measures, virus biological characteristics and community characteristics. The paper also finds that infections are mostly based on strong ties. Therefore, for suspected infection, in-house quarantine is not effective since cluster infection may occur in the family. Self-isolation in a hospital or hotel room for suspected infection is a better way. However, the effectiveness of this quarantine policy depends on the capacity of isolation place. Moreover, good hygiene cognitions and habits for family members should be emphasized.

Few existing studies on COVID-19 analyzed public case reports from the perspective of contact network. Furthermore, the phase change structure of the dynamic network is very different from the static network^23, 24^. The dynamic change of the network has an important impact on the risk of infection. But the research on dynamic contact networks is still less. This research enriches existing research and helps to better understand the dynamic social network transmission of COVID-19. This study also confirms that case reports contain a wealth of information for text mining, and the contact network can be constructed through text analysis technology, which is beneficial to epidemic prevention and control.

Due to time and data limitations, there are still some shortcomings in this study. First, case analysis is limited to Shaanxi and there is no effective way to present a virus contact network across regions by far. Second, the number of cases in this study is relatively limited. Third, changes in cognitions of the virus will undoubtedly change the network structure of virus transmission, but this study also lacks the corresponding attitude data of individuals. Finally, the contact network only can capture part of the dynamic transmission process of COVID-19. Merging more data with the network data, such as virus biological data, community characteristics and capacity of quarantine place, can help us better understand the virus transmission process. I hope that there will be more studies on the contact network of COVID-19 in the future based on detailed epidemic data.

## Conclusion

Viruses spread through human social networks. However, existing studies are less likely to analyze the contact network of COVID-19 and less likely to dig deeper into the information behind the public case reports. In this study, by text mining the case reports of the early 2020 outbreak in Shaanxi, China, I mined the basic statistics of patients and constructed a dynamic contact network to analyze different virus transmission routes: strong ties, weak ties, and strangers.

## Data Availability

All data and code are on an OSF data repository, see https://osf.io/h9qmu/. I made a video to depict the dynamic change of the contact network from January 23th to February 16th, see https://youtu.be/JtU1sfgjup8. All raw data (in Chinese) is available on the official website of Health Committee of Shaanxi Province of China, see http://sxwjw.shaanxi.gov.cn/col/col9/index.html. All data has been anonymized.

## References

[1] Munster VJ, Koopmans M, van Doremalen N, van Riel D, de Wit E (2020). A novel coronavirus emerging in china—key questions for impact assessment. New Engl. J. Med..

[2] Chan JF (2020). A familial cluster of pneumonia associated with the 2019 novel coronavirus indicating person-to-person transmission: a study of a family cluster. Lancet.

[3] Zhu, N. et al. A novel coronavirus from patients with pneumonia in China, 2019. *New Engl. J. Med.* (2020). 10.1056/NEJMoa2001017 PMC7092803 31978945

[4] Li, Q. et al. Early transmission dynamics in wuhan, China, of novel coronavirus: infected pneumonia. *New Engl. J. Med.* (2020). 10.1056/NEJMoa2001316 PMC7121484 31995857

[5] Phan LT (2020). Importation and human-to-human transmission of a novel coronavirus in Vietnam. New Engl. J. Med..

[6] Chinazzi M (2020). The effect of travel restrictions on the spread of the 2019 novel coronavirus (COVID-19) outbreak. Science.

[7] Wang C, Horby PW, Hayden FG, Gao GF (2020). A novel coronavirus outbreak of global health concern. Lancet.

[8] Chang D (2020). Epidemiologic and clinical characteristics of novel coronavirus infections involving 13 patients outside Wuhan, China. JAMA.

[9] Bottemanne, H., Morlaàs, O., Fossati, P. & Schmidt, L. Does the coronavirus epidemic take advantage of human optimism bias? *Front. Psychol*. **11** (2020). 10.3389/fpsyg.2020.02001 PMC7479219 32982839

[10] Bearman PS, Moody J, Stovel K (2004). Chains of affection: the structure of adolescent romantic and sexual networks. Am. J. Sociol..

[11] Marvel, S. A., Martin, T., Doering, C. R., Lusseau, D. & Newman, M. E. *The Small-World Effect is a Modern Phenomenon*. https://arxiv.org/abs/1310.2636 (2013).

[12] Salathé M (2010). A high-resolution human contact network for infectious disease transmission. Proc. Natl. Acad. Sci..

[13] Jaffe HW (2008). The early days of the HIV-AIDS epidemic in the USA. Nat. Immunol..

[14] Kuperman M, Abramson G (2001). Small world effect in an epidemiological model. Phys. Rev. Lett..

[15] Watts DJ, Strogatz SH (1998). Collective dynamics of ‘small-world’networks. Nature.

[16] Eubank S (2004). Modelling disease outbreaks in realistic urban social networks. Nature.

[17] Meyers LA, Pourbohloul B, Newman ME, Skowronski DM, Brunham RC (2005). Network theory and SARS: predicting outbreak diversity. J. Theor. Biol..

[18] Chen S (2014). Highly dynamic animal contact network and implications on disease transmission. Sci. Rep. UK.

[19] Huang C (2020). Clinical features of patients infected with 2019 novel coronavirus in Wuhan, China. Lancet.

[20] Chen N (2020). Epidemiological and clinical characteristics of 99 cases of 2019 novel coronavirus pneumonia in Wuhan, China: a descriptive study. Lancet.

[21] Tian FF, Lin N (2016). Weak ties, strong ties, and job mobility in Urban China: 1978–2008. Soc. Netw..

[22] Lau MS (2017). Spatial and temporal dynamics of superspreading events in the 2014–2015 West Africa ebola epidemic. Proc. Natl. Acad. Sci..

[23] Armbruster B, Wang L, Morris M (2017). Forward reachable sets: analytically derived properties of connected components for dynamic networks. Netw. Sci..

[24] Onaga T, Gleeson JP, Masuda N (2017). Concurrency-induced transitions in epidemic dynamics on temporal networks. Phys. Rev. Lett..

